# Supplementary figures and images for: Phylogenomics of darkling beetles (Coleoptera: Tenebrionidae) from the Atacama Desert
Source: PeerJ. 2023 Feb 23;11:e14848. doi: 10.7717/peerj.14848 (PMC9968461; doi:10.7717/peerj.14848)

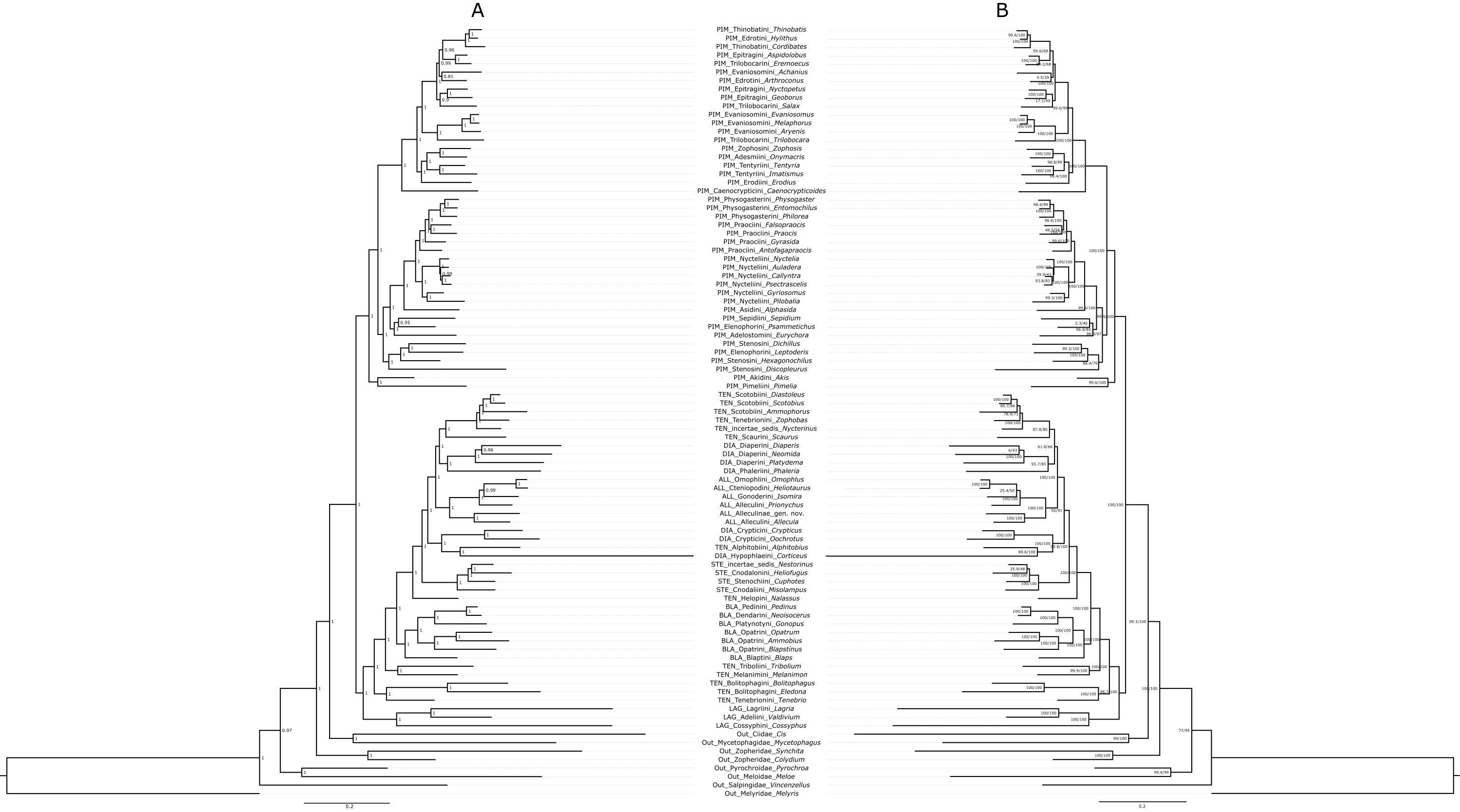

Supplement: Supplemental Information 3 — Phylogenetic trees resulting from BI and ML analyses of the partitioned 34 neuropeptide and neuropeptide–like precursors from 83 genera of Tenebrionidae, including the 30 genera from the Atacama Desert. (A) BI tree with posterior probability values for each branch. (B) ML tree with bootstrap support values for each branch (SH-aLRT test / UFBoot). [file peerj-11-14848-s003.png]

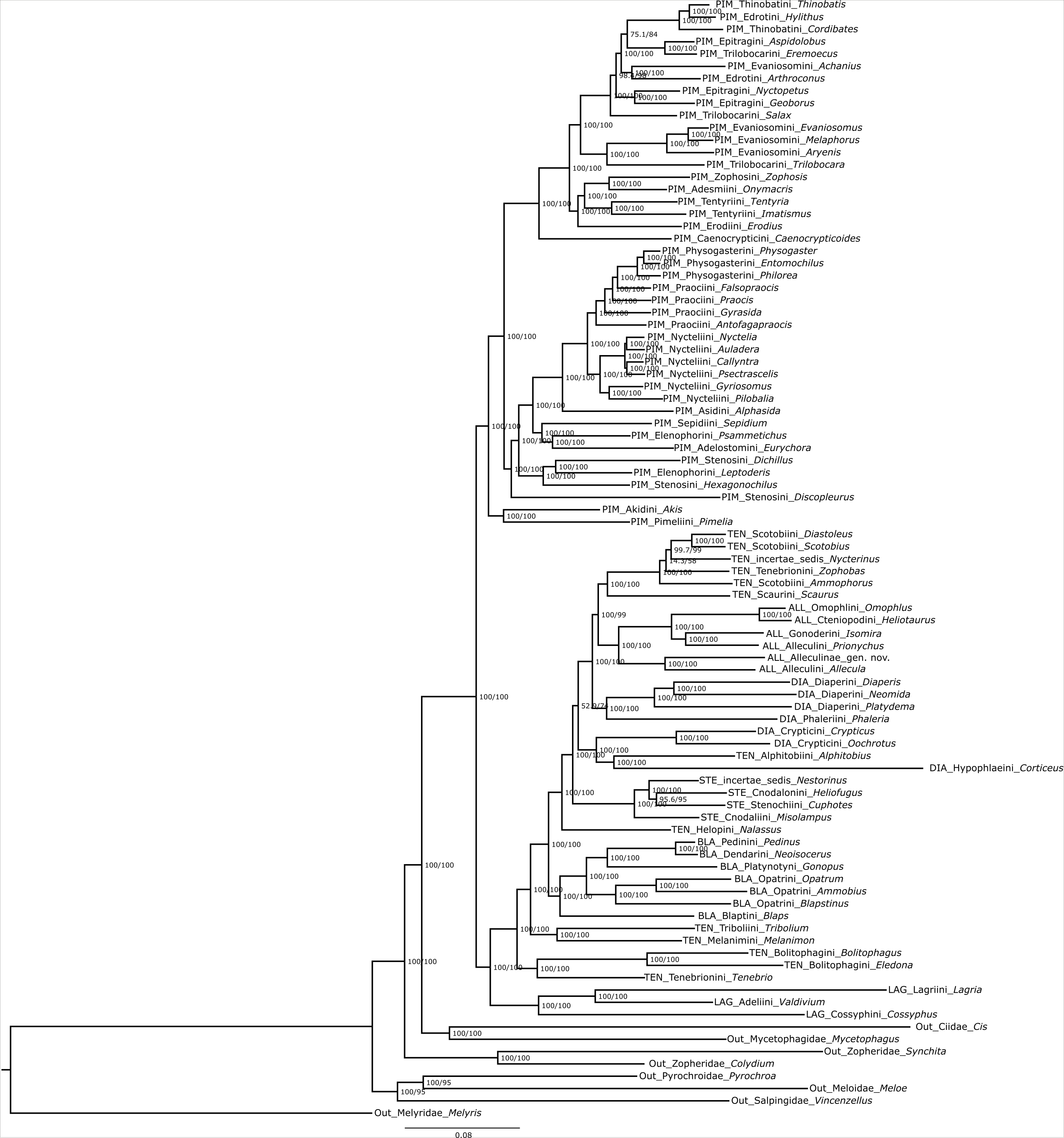

Supplement: Supplemental Information 4 — ML tree of the partitioned amino acid supermatrix of 1742 OGs. Each node with branch support values SH-like / UFBoot. [file peerj-11-14848-s004.png]
